# Role of Self-Trapped Excitons in the Broadband Emission of Lead-Free Perovskite-Inspired Cu_2_AgBiI_6_

**DOI:** 10.1021/acs.jpclett.3c00439

**Published:** 2023-04-28

**Authors:** G. Krishnamurthy Grandhi, Rakesh Dhama, Noolu Srinivasa Manikanta Viswanath, Ekaterina S. Lisitsyna, Basheer Al-Anesi, Jayanta Dana, Vipinraj Sugathan, Humeyra Caglayan, Paola Vivo

**Affiliations:** † Hybrid Solar Cells, Faculty of Engineering and Natural Sciences, Tampere University, P.O. Box 541, FI-33014 Tampere University, Finland; ‡ Faculty of Engineering and Natural Sciences, Tampere University, 33720 Tampere, Finland; § Division of Materials Science and Engineering, Hanyang University, 222 Wangsimni-ro, Seongdong-gu, Seoul 04763, Republic of Korea; ∥ Faculty of Engineering and Natural Sciences, Tampere University, Korkeakoulunkatu 8, 33720 Tampere, Finland

## Abstract

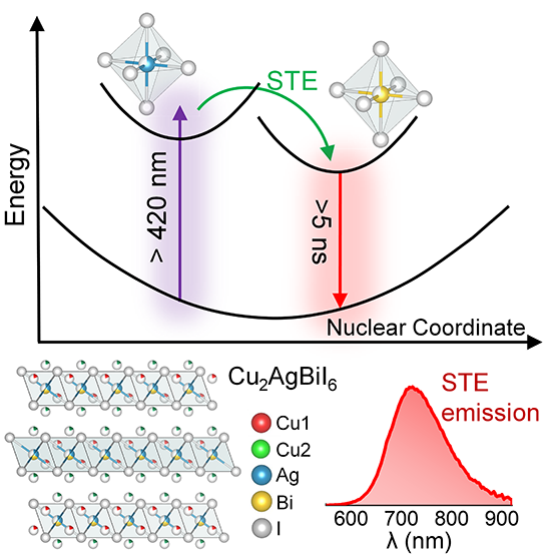

The perovskite-inspired Cu_2_AgBiI_6_ (CABI) absorber shows promise for low-toxicity indoor photovoltaics. However, the carrier self-trapping in this material limits its photovoltaic performance. Herein, we examine the self-trapping mechanism in CABI by analyzing the excited-state dynamics of its absorption band at 425 nm, which is responsible for the self-trapped exciton emission, using a combination of photoluminescence and ultrafast transient absorption spectroscopies. Photoexcitation in CABI rapidly generates charge carriers in the silver iodide lattice sites, which localize into the self-trapped states and luminesce. Furthermore, a Cu–Ag–I-rich phase that exhibits similar spectral responses as CABI is synthesized, and a comprehensive structural and photophysical study of this phase provides insights into the nature of the excited states of CABI. Overall, this work explains the origin of self-trapping in CABI. This understanding will play a crucial role in optimizing its optoelectronic properties. It also encourages compositional engineering as the key to suppressing self-trapping in CABI.

Lead-free perovskite-inspired materials (PIMs) with wide band gap can offer a sustainable solution for the development of efficient tandem solar cells and indoor photovoltaics (IPVs).^1−4^ Among the PIMs explored for IPVs, the bismuth(III) (Bi^3+^)-based^5^ Cu_2_AgBiI_6_ (CABI) stands out as a promising absorber, owing to its high air-stability^1,4^ and, more importantly, its direct band gap of ∼2 eV that can theoretically lead to an ultimate indoor power conversion efficiency (PCE(i)) up to 60%.^2,4^ However, the PCE(i) of the IPVs based on this emerging material is still far from the theoretical limit, with the highest value being 5.52% at 1000 lx illumination.^6^ The major performance-limiting parameters of CABI-based photovoltaics are the very low open-circuit voltage (*V*_OC_) and short-circuit current density (*J*_SC_) values.^1,4^ In addition to the well-established role of intrinsic vacancies and defects,^7,8^ another notable fundamental process that contributes to large voltage and current losses in photovoltaic devices based on low-dimensional metal halides is carrier self-trapping.^8,9^ For CABI PIM, Buizza et al. demonstrated a picosecond charge-carrier localization and the subsequent broadband self-trapped exciton (STE) emission through an extensive optical-pump terahertz-probe spectroscopy and temperature-dependent photoluminescence (PL) study.^10^ However, in a previous report we have observed an energy mismatch of ∼80 meV between the PL excitation (PLE) peak and the first excitonic absorption maximum of CABI,^4^ in contrast with the closely spaced absorption and PLE spectra of well-studied STE-emitting materials such as Cs_2_Ag_*x*_Na_1–*x*_InCl_6_.^11,12^ In addition, the PLE peak of CABI matched that of a high-energy absorption peak at 420–425 nm, which was temporarily assigned to an unidentified impurity.^4^ These findings suggest a complex STE emission mechanism occurring in CABI. Very recently, by coalloying antimony(III) (Sb^3+^) with Bi^3+^ in CABI, we successfully quenched the STE emission of CABI, in turn significantly boosting the *J*_SC_ of CABI-Sb IPVs, which achieved a bold PCE(i) of nearly 10%.^13^ This clearly suggests that the suppression of the self-trapping of the charge carriers is crucial to enhance the performance of CABI-based devices. Nevertheless, the understanding of the STE emission in CABI is still limited and the origin of the PLE spectrum of CABI is yet unknown. Understanding the role and the nature of the above-mentioned absorbing species at 425 nm would not only shine light on the emission mechanism and the origin of the carrier self-trapping in CABI but would enable further optimization of this material, which has shown proven potential in photovoltaics^1,4,6,14^ and beyond.^15^

In this work, we investigate the excited-state charge-carrier dynamics in CABI using ultrafast transient absorption (TA) spectroscopy. We suggest a plausible mechanism for the STE emission, wherein the charge carriers from a high-energy excited state rapidly transfer to a low-lying self-trapped state and radiatively relax to the ground state. Thermal annealing of CABI film beyond its decomposition point produces a Cu–Ag–I-rich phase, which possesses strong absorption characteristics in a similar wavelength range as the 425 nm band of CABI. However, the emission characteristics of the CABI and Cu–Ag–I rich phases differ. The comparable TA dynamics of the high-energy absorbing band and the PLE spectra of both CABI and Cu–Ag–I-rich films reveal that a similar initial excited state is present in both materials. This allows us to hypothesize that the broad band STE emission of CABI involves the localization of the photoexcited carriers from the silver sites of the lattice to the STE states. Our findings will promote in-depth knowledge of the excited-state charge-carrier localization in the wide silver–bismuth PIM family.

The two-dimensional CABI structure comprises edge-sharing Ag^+^ and Bi^3+^ iodide octahedra and Cu–I tetrahedra, which crystallizes in a *R*3̅*m* space group (Figure 1a). The alternating layers of octahedral sites of the structural framework of CABI is a highly disordered network with the cubic close-packed iodide sublattice partially occupied by Bi or Ag. We fabricated CABI films for structural, morphological, and photophysical characterizations through spin-coating and then annealing in the air in two steps (see the Supporting Information for experimental details).^1,4^ Their structural, absorption, and PL properties are consistent with our earlier report^4^ (see Figures S1 and 1b,c). Briefly, CABI exhibits two absorption bands with maxima at 425 and 580 nm (Figure 1b). CABI exhibits a broad emission covering 600–900 nm and peaking at 720 nm (1.72 eV), with a corresponding full width at half-maximum (fwhm) of ∼310 meV. The PL peak wavelength of 720 nm (1.72 eV) closely matches (negligible difference of 10 meV) that of the CABI films (i.e., 725 nm (1.71 eV)) studied earlier.^1^ The PL quantum yield (PLQY) is very low (∼0.1%), consistent with the weak emission intensities reported for CABI earlier.^1,4,14^ Typically, this is the case with two-dimensional metal halide perovskites,^16^ unlike zero-dimensional metal halide perovskites.^17^ This is because the broadband STE emission is more dominant in metal halide perovskites with low structural and electronic dimensionalities,^18^ in which the charge carrier–phonon coupling is very strong. In addition, defect-related nonradiative recombination also contributes to the low PLQY of the STE emission. Another major nonradiative recombination pathway for STEs contributing to the very low PLQY of CABI arises from the lattice vibrations.^10^ The STE emission intensity of CABI gradually increased with reducing the temperature, suggesting the suppression of lattice vibration-mediated nonradiative recombination of STEs. Temperature-dependent STE emission decay of CABI confirmed this—the average lifetime of the emission increased by 2 orders of magnitude when the temperature was reduced from room temperature to 4 K.^10^ The substantially lowered (i.e., by 2 orders of magnitude) emission lifetime suggests the loss of the majority of the STEs before their radiative recombination through the vibrations (as well as defect-related recombination) at room temperature, thus leading to a very low PLQY. Similarly, various 2D metal halides (e.g., Cs_3_Bi_2_I_9_, Cs_3_Sb_2_I_9_, and Rb_3_Sb_2_I_9_, and (Cs/MA/FA)_3_Sb_2_I_9_) exhibit broad and very weak STE emission intensities despite the strong charge carrier self-trapping.^19−21^ Therefore, regardless of the low PLQY (which only provides information about the radiative recombination of the STEs but not the total number of STEs formed), understanding the STE process in CABI, a lead-free PIM for photovoltaic and other optoelectronic applications, is of utmost significance.

**Figure 1:**
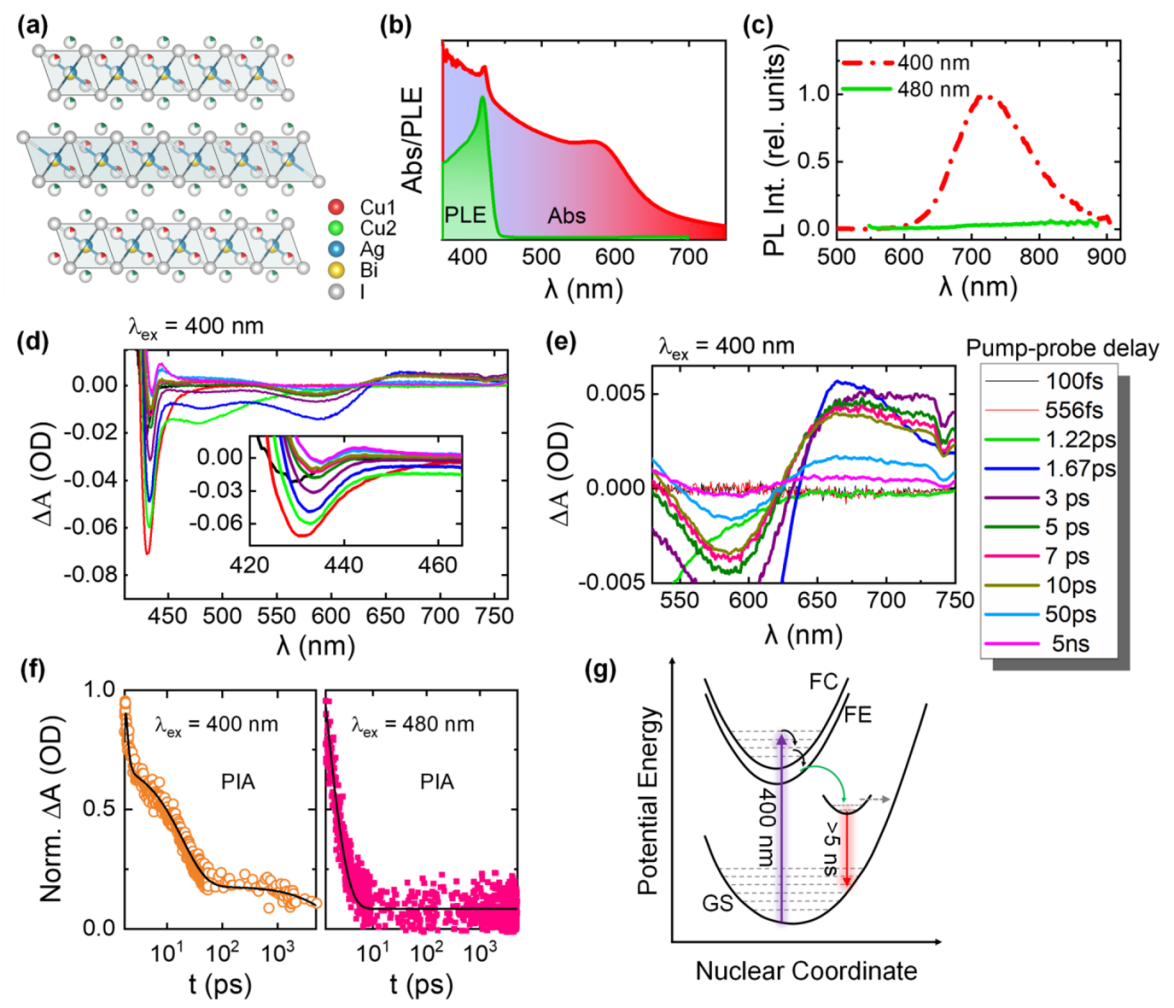
(a) Crystal structure of Cu_2_AgBiI_6_ (CABI). (b) Absorption and PLE spectra of a CABI film on glass. (c) PL spectra of the CABI film excited at 400 and 480 nm. TA spectra (pump wavelength is 400 nm) of the CABI film with varying pump–probe delay in the (d) 410–760 nm and (e) 530–760 nm ranges. (f) PIA decay curves collected at 670–680 nm under the 400 and 480 nm pump excitations. (g) Schematic of the adiabatic potential energy curves of the ground state (GS), free carrier (FE), free exciton (FE), and a low-energy excited state (i.e., STE state) along with a horizontal dashed arrow indicating the possible nonradiative process in a configuration space, under 400 nm photoexcitation. Typically, the energy difference between FC and FE is equal to the exciton binding energy (*E*_b_), i.e., FC – *E*_b_ = FE.^23^

The PLE spectrum of CABI exhibits a distinct peak nearly matching its 425 nm absorption band, and no luminescence was detected at an excitation below the onset energy of the 425 nm feature (Figure 1c). Therefore, the 425 nm absorption band holds the key for the observed emission. The absence of band edge emission under 480 nm excitation could be mainly due to the predominant nonradiative relaxation of the excited charge carriers through a high concentration of defects (both bulk- and surface-related) in the wide band gap CABI. Sub-band gap states have been identified experimentally in CABI.^1^ Subpar solar cell performance of the CABI has been attributed to the poor radiative recombination in this material.^1,4,14^

We characterized the excited-state charge-carrier dynamics in CABI, including the STE formation, through ultrafast TA measurements. Figure 1d shows the TA spectra of CABI in the 420–750 nm probe range, excited with 400 nm pump pulses, i.e., well above the material’s band gap. Such high excitation energy usually pumps the electrons into the higher excited states (e.g., free-carrier state) rather than the excitonic one. The photo bleaching signal centered at ∼590 nm can be attributed to the ground-state depopulation or bleaching (GSB), as its peak position closely matches the first excitonic peak of CABI (Figure 1d,e). The bleach band centered at ∼470 nm can be attributed the GSB of the second excitonic peak of CABI. A broad positive photoinduced absorption (PIA) band centered at 670 nm spreads out from 620 nm up to beyond 760 nm (Figure 1e). The broad PIA band first appears approximately 1 ps after the photoexcitation, and this time scale is comparable to the previously observed picosecond carrier trapping or small polaron formation in CABI.^10^ The broadening of the PIA band as the delay time changes from 1.67 ps to ≥3 ps (Figure 1e) can be attributed to the slightly enhanced distortion of the excited-state species at longer delay times. The charges from this low-lying PIA state relax down to the ground state on the time scale beyond our instrument’s measurement range of 5 ns (Figure 1f). The PIA decay was fitted with a triexponential function. The resultant ultrafast (730 fs) and fast components (19 ps) can be ascribed to nonradiative recombination, whereas the slow decay channel (5.7 ns) represents the radiative recombination (see Figure 1g).^11,22^ The corresponding amplitudes are 47 ± 3% (730 fs), 41 ± 2% (19 ps), and 12 ± 2% (5.7 ns). On the other hand, the decay takes place mostly within the first 10 ps (Figure 1f) under near band-edge excitation at 480 nm, well below the energy of the 425 nm absorption band, where no emission was detected (Figure 1c). The decay curve was fitted with a biexponential function, with the corresponding time constants being 780 fs (amplitude = 79 ± 4%) and 2 ps (amplitude = 21 ± 2%).

The radiative relaxation of the carriers (slow decay of the PIA band) and the presence of luminescence under 400 nm excitation hints at the STE emission.^10,11,22^ However, the STE emission process involves ultrafast transfer (1–2 ps) of the carriers from a higher photoexcited state into an STE state.^22,24^ Since the luminescence of CABI occurs through the photoexcitation of the 425 nm absorption feature (see Figure 1b), we studied its excited-state carrier dynamics. A photobleaching (PB) band centered at 428–430 nm that appears almost instantaneously (100 fs) (see the inset of Figure 1d) could be attributed to the 425 nm absorption feature. On the other hand, the PIA band appears after nearly 50% of the PB band is recovered (Figure S2). This might suggest an energy or charge transfer from the species corresponding to the 425 nm absorption band to the STE states of CABI, resulting in the broad emission centered at 720 nm. It is thus necessary to recognize the origin of the 425 nm absorption band and then study its optical properties to understand the STE process in CABI.

Apart from Cu_2_BiI_5_^1^ or Ag–Bi–I and Cu–Ag–I-based impurities,^25^ very small quantities of AgI or BiI_3_ may coexist with CABI. While the AgI is nonemissive,^26,27^ the PLE spectrum and TA dynamics of BiI_3_ (Figure S3) suggest that it is not the source of emission. We employed fluorescence lifetime imaging microscopy (FLIM) to examine the coexistence of luminescence impurities within CABI.^28^ The FLIM image of CABI film under 405 nm excitation (see Figure 2a) comprises two distinct regions based on their luminescence lifetimes, with the “boundary” regions of interest (ROI) exhibiting a higher lifetime of 44 ns than the 27 ns of “center” ROI (Figure 2b). The FLIM images (Figure 2a,c) also comprise nearly nonluminescent (dark) domains. The comparison of the FLIM image (Figure 2c) with the SEM image (Figure 2d) hints that the nonluminescent areas cannot be solely attributed to the pin holes or gaps in the film. The FLIM images, overall, suggest an intermittent luminescence behavior for the CABI film, but they do not provide any conclusive evidence of the emission from tiny impurity quantities (undetected in XRD). A separate follow-up study will be necessary to understand the emission inhomogeneity of the CABI films.

**Figure 2:**
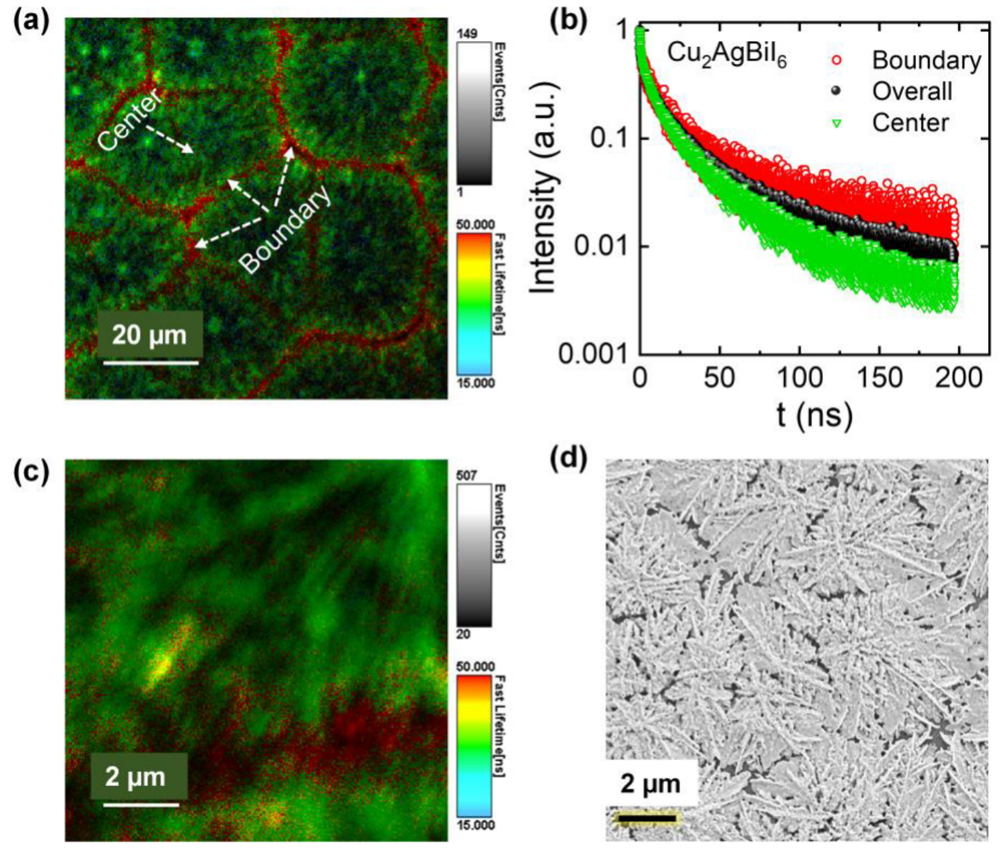
(a) FLIM image of a CABI film. (b) The emission decay curves of the different regions of interest (ROI) highlighted in panel a. The average lifetime values for the boundary, center, and overall ROI are 44, 27, and 32 ns, respectively (see the Experimental Section in the Supporting Information for the fitting details). (c) A high-resolution FLIM image of the CABI film. (d) SEM image of the CABI film.

We monitored the variation in structural and optical properties of Cu–Ag–Bi–I films fabricated at different annealing temperatures in the second step of the fabrication process (see the Experimental Section in the Supporting Information). Figure S4 shows the evolution of XRD patterns, absorption, PL, and PLE spectra of Cu–Ag–Bi–I films with increasing the annealing temperature (during fabrication) from 75 to 330 °C. The XRD patterns of the films remain similar and retain the CABI structure up to 230 °C, whereas they decompose into a new crystal phase beyond 250 °C and maintain the same XRD pattern up to 290 °C (Figure S4a). This structural change is evident from the disappearance of one of the characteristic XRD peaks of the CABI phase at ∼12.8°. Figure 3a shows the relative change in the Bi content for the films annealed in the 75–330 °C range. The amount of Bi in the films (detected using scanning electron microscopy-energy dispersive X-ray spectroscopy (SEM-EDS)) remains unchanged up to 200 °C, consistent with their stable structural characteristics; however, a 30% deficiency of Bi was detected at 230 °C annealing. Almost Bi-free films were obtained at 260–290 °C, which is consistent with the decomposition temperature of ≥250 °C of CABI.^14^ The increased number of gaps in the films (Figure 3b) as the temperature increased from 200 to 230 °C and then to 260–290 °C can be attributed to the evaporation of Bi with temperature, as evident from the Bi trend in Figure 3a. The structural transformation associated with the Bi evaporation also resulted in the color change of the films from dark red (75–200 °C) to transparent yellow (260–290 °C), as shown in Figure 3b. The elemental composition of the Cu–Ag–I rich yellow-colored films is Cu_2.2_Ag_2.0_Bi_0.16_I_4.0_ (Cu_2_Ag_2_I_4_:Bi). Rietveld refinement reveals that the XRD pattern (see Figure 3c and Tables S1 and S2) of Cu_2_Ag_2_I_4_:Bi shows a full convergence (χ^2^ = 1.2 and *R*_wp_ = 1.8%) with the basic model of Cu_2_Ag_2_I_4_ (space group: *F*4̅3*m*), suggesting the absence of impurity phases. In addition, Figure S5 compares the XRD pattern of Cu_2_Ag_2_I_4_:Bi film with the standard patterns of Cu_2_Ag_2_I_4_ and Cu_2_AgBiI_6_. It is evident from the figure that the XRD pattern of Cu_2_Ag_2_I_4_:Bi does not match that of Cu_2_AgBiI_6_. For instance, the peak at ∼13° present in the XRD patterns of Cu_2_AgBiI_6_ standard and Cu_2_AgBiI_6_ films is absent in that of Cu_2_Ag_2_I_4_:Bi film. The metal cations in the Cu_2_Ag_2_I_4_ structure are bonded to four equivalent I^–^ to form corner-sharing metal iodide tetrahedra (Figure 3d).^29^ The deviation in the occupancies of Ag and I from their respective ideal values of 0.5 and 1.0 suggests the existence of both silver and iodine vacancies in the material. A very high annealing temperature of 330 °C led to the formation of a nonemissive Cu_0.58_Ag_2.0_Bi_0.12_I_2.8_ film (Figure S4c).

**Figure 3:**
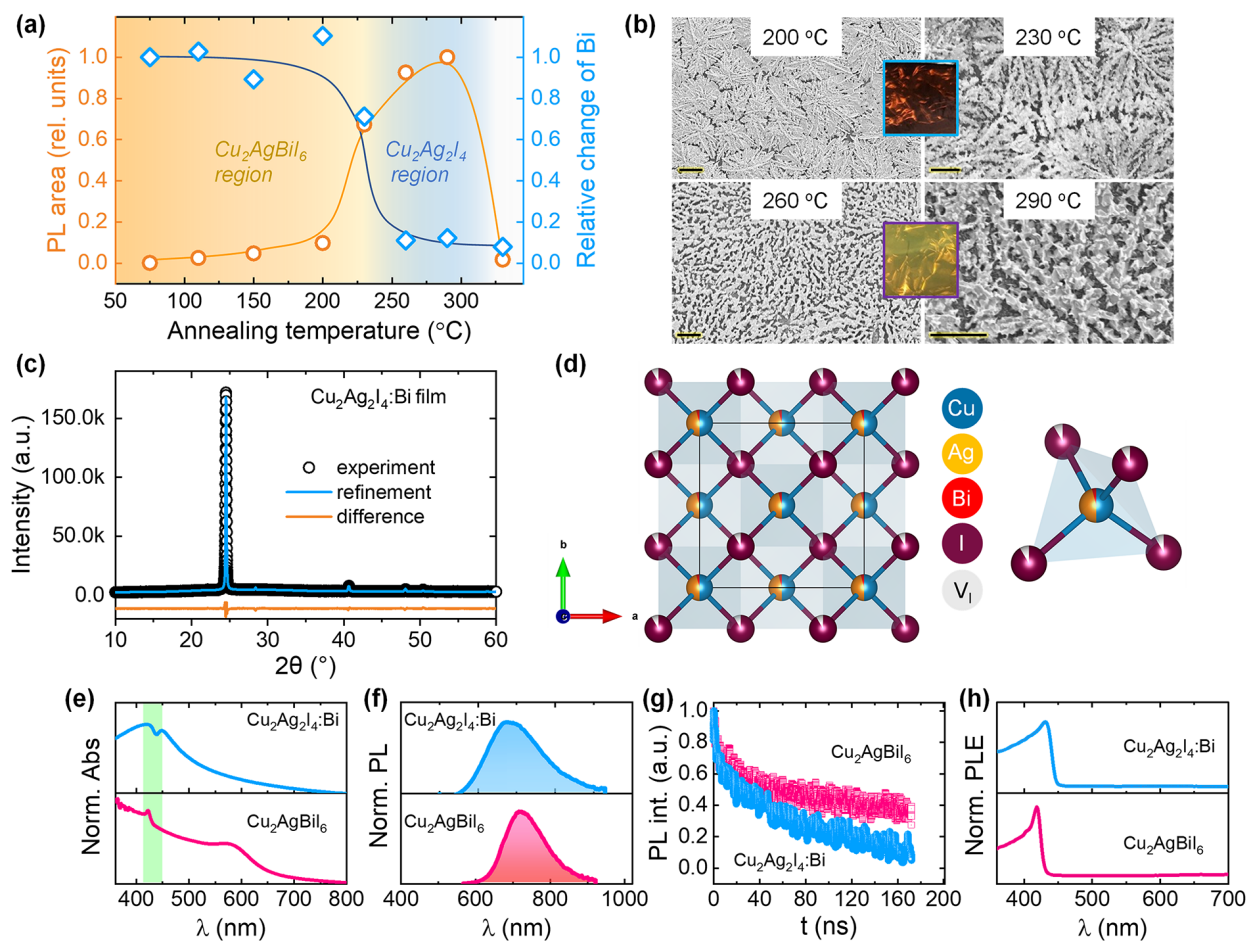
(a) Variations of integrated PL intensity and relative change in Bi quantity (obtained from SEM-EDS) of Cu–Ag–Bi–I films with the annealing temperature. (b) SEM images of Cu–Ag–Bi–I films fabricated at 200, 230, 260, and 290 °C. Scale bar is 2 μm. The insets display the photographs of CABI (dark red) and Cu_2_Ag_2_I_4_:Bi (yellow) films under room light. (c) Experiment (black open circles), refinement (blue solid line), and difference (orange solid line) profiles obtained after full-pattern Rietveld refinement. (d) Refined crystal structure of Cu_2_Ag_2_I_4_:Bi along with the metal iodide tetrahedral representation. (e) Normalized absorption, (f) normalized PL spectra (λ_ex_ = 400 nm), (g) TRPL decay curves (λ_ex_ = 405 nm), and (h) normalized PLE spectra of CABI and Cu_2_Ag_2_I_4_:Bi films.

The Cu_2_Ag_2_I_4_:Bi film absorbs strongly in the 420–450 nm region (Figure 3e), which is comparable to the absorption characteristics of AgI–CuI films reported by Cha et al.^30^ This suggests that the residual Bi likely does not contribute significantly to the absorption properties of Cu_2_Ag_2_I_4_:Bi. Also, peak splitting seems to cause the apparent hole in the 420–450 nm region of the absorption spectrum. Similar to the AgI–CuI films,^30^ the broad absorption tail of the Cu_2_Ag_2_I_4_:Bi film in the visible region can partially be attributed to the scattering associated with its rough film morphology (see Figure 3b). The PL spectrum of Cu_2_Ag_2_I_4_:Bi is broader (550–920 nm) and blue-shifted (centered at 700 nm) by 20 nm compared to CABI (Figure 3f). The PLQY of Cu_2_Ag_2_I_4_:Bi is ∼0.2%. The time-resolved PL (TRPL) decay curves of both materials are also not identical (Figure 3g). On the contrary, the corresponding PLE spectra of CABI and Cu_2_Ag_2_I_4_:Bi (Figure 3h) possess similar spectral shapes and peak wavelengths (difference of ±10 nm), in line with their closely matching absorption behavior in the shaded region of Figure 3e, thereby suggesting a similar initial photoexcited state.

The absorption spectrum of Cu_2_Ag_2_I_4_:Bi comprises two absorption peaks, peak-1 (λ_max_ ≈ 422 nm) and peak-2 (λ_max_ ≈ 451 nm), as shown in Figure S6. The peak-1 (λ_max_ ≈ 422 nm) closely matches the PLE peak wavelength (λ_max_ ≈ 429 nm). Consequently, most of the PLE spectrum falls in the peak-1 region, whereas the PLE intensities are very low in the peak-2 region. This suggests that photoexcitation by energies in the peak-1 region generates free charge-carriers that relax to the green potential of the scheme in Figure 4d. On the other hand, in the peak-2 region, i.e., energies below those of peak-1, the photoexcited carriers mostly relax through nonradiative recombination pathways. Since peak-2 is the lowest-energy absorption band, it can likely be related to the band edge absorption. The electronic band structure calculations may provide insights into the nature of the band gap and the origin of the two absorption bands.

**Figure 4:**
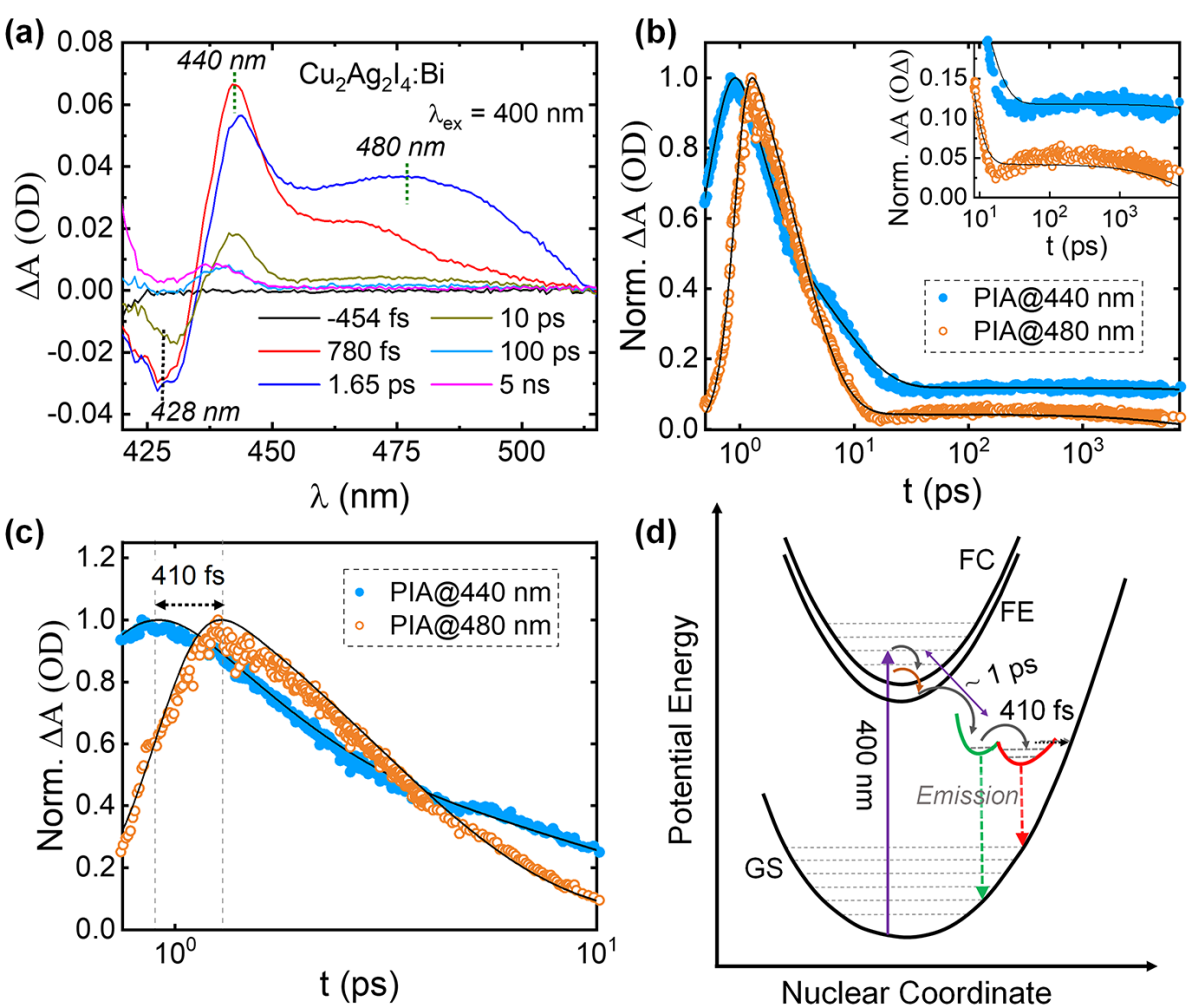
(a) TA spectra (in the 420–550 nm range) of the Cu_2_Ag_2_I_4_:Bi film excited at 400 nm. (b) The decay curves of PIA peaks at 440 and 480 nm. The inset highlights the slow components (which correspond to radiative recombination) of the two decay curves. (c) The decay curves of PIA peaks at 440 and 480 nm up to 10 ps. (d) Schematic of the adiabatic potential energy curves of the ground state (GS), free carrier (FE), free exciton (FE), and dual trapped emissive excited states in a configuration space. The vertical dashed arrows and the horizontal dashed arrow indicate the radiative (emission) and nonradiative processes, respectively, under 400 nm photo excitation.

To probe into the comparable PLE spectra of Cu_2_Ag_2_I_4_:Bi compared to CABI, its TA spectra under 400 nm excitation were collected (Figure 4a). A PB band is identified at 428 nm, which matches the peak wavelength of the PB band of CABI (inset of Figure 1d). This band depopulates in <1 ps and exhibits similar recovery dynamics as in the case of CABI (Figure S7), consistent with the closely matching PLE spectra of Cu_2_Ag_2_I_4_:Bi and CABI (Figure 3h). Figure 4a also shows a broad PIA signal composed of two peaks at 440 and 480 nm. The decays of the 440 and 480 nm PIA bands (Figure 4b) were fitted to tri- and biexponential decay functions, respectively. The resultant decay times are ∼1 ps, 8 ps, and >7 ns (440 nm PIA band) and 3 ps and >7 ns (480 nm PIA band). The ultrafast (∼1 ps) component provides information about the charge carrier trapping time. This time scale is within the typical range observed for other metal halides.^16,31^ The slow decay (>7 ns) components (which refer to radiative recombination) of the two PIA bands may correspond to two long-lived emissive trapped states that contribute to the broad PL of Cu_2_Ag_2_I_4_:Bi. The inhomogeneously broadened PL spectrum can be deconvoluted with two Gaussian profiles (see Figure S8), which further suggests the existence of more than a single emissive excited state.^16,32^ The delayed turn-on time (by 410 fs) of the decay of the low-energy 480 nm PIA band (Figure 4c) indicates that it belongs to a deeper trapped emissive state.^16,31^ The amplitude of the 480 nm PIA band increases at the cost of a decrease in that of the 440 nm one when the pump–probe delay time increases from 780 fs to 1.65 ps. The ultrafast (∼1 ps) channel of the 440 nm PIA decay may also partially be attributed to the cascade movement of the excited carriers from the shallow (which corresponds to the 440 nm PIA band) to the deeper trapped states (which corresponds to 480 nm PIA band), as shown in Figure 4d. A previous report on 2D lead halide hybrid perovskites also demonstrated shuttling of the excited carriers from shallow to deeper states on a similar time scale as 410 fs, i.e., a few hundred femtoseconds.^16^ Permanent lattice vacancies or correlated self-trapping carrier states have been proposed to contribute toward multiple emissive trapped state generation.^16,33^ The cation and anion vacancies in the crystal structure of Cu_2_Ag_2_I_4_:Bi (see Figure 3d and Table S2) likely contribute to traps of variable depths, namely, shallow and deep traps in the band structure.^34^ We propose that both Ag^+^ and I^–^ defects contribute to the two emitting states shown in Figure 4d. This can be supported by the observations of Annadi et al. on Ag_*x*_Cu_1–*x*_I alloy samples, in which Ag and I defect levels act as two emitting states with distinct energy positions.^35^ In another study, Wei et al. proposed radiative recombination between the trapped carriers in interstitial silver ion vacancies in the silver-rich regions and the vacancy-compensated ions, leading to a broad emission covering 500–900 nm for CuI/AgI glass system.^27^ Further, the strong covalent nature of Ag–I bonds^27,36^ might inhibit the self-trapping through the Ag–I polyhedral distortion, in contrast to the facile STE formation through the distorted AgCl_6_ in Cs_2_AgInCl_6_^11^ and AgCl crystals.^37^ The broad emission of Cu_2_Ag_2_I_4_:Bi arising from dual emissive trapped states could be a consequence of more extrinsic, that is, defect/vacancy-mediated than intrinsic self-trapping.^16,33,38,39^ The influence of the lattice defects on local structural perturbation^40^ in the excited state can be further verified using ultrafast X-ray absorption techniques.^41^ The broad PB band that covers full visible wavelengths (Figure S9) can be ascribed to both the stimulated emission and the broad absorption tail (Figure 3e) resulting from the structural disorder or defects.^42^ Neither charge carrier generation nor emission takes place in Cu_2_Ag_2_I_4_:Bi under 480 nm photoexcitation (since no TA and PL signals were detected).

The similarity of both the high-energy PB band recovery dynamics (TA spectra) and the PLE spectra between CABI and Cu_2_Ag_2_I_4_:Bi may indicate that silver-containing regions of the lattice contribute to the initial photoexcited state (that is, the 425 nm absorption band) of CABI. Nevertheless, the nature of emission pathways of CABI and Cu_2_Ag_2_I_4_:Bi are different, namely, exciton or free carrier transfer (CABI) and vacancy-assisted trapping (Cu_2_Ag_2_I_4_:Bi). The 425 nm absorption feature of CABI can be attributed to the silver iodide lattice sites as supported by the matching absorption characteristics of AgI crystals measured at 295 K^43^ and the 415–425 nm band observed in the absorption spectrum of Ag_3_BiI_6_ arising from AgI impurities.^44^ We and others^1,10,14^ thus far did not find any AgI impurity in CABI films (see Figure S1). Moreover, AgI is known to be nonemissive at room temperature.^43^ Similar to the case of CABI, Sharma et al. attributed an additional peak observed at higher energy than the excitonic peak in the absorption spectra of Rb_4_Ag_2_BiBr_9_ to the AgBr_5_ units of the material.^45^ In addition to the increasing integrated PL intensity (Figure 3a), the fwhm of the emission rises with decreasing Bi quantity in the Cu–Ag–Bi–I films—the fwhm values are 0.3 and 0.45 eV for CABI and Cu_2_Ag_2_I_4_:Bi, respectively. This suggests the involvement of Bi lattice sites in CABI emission. This is in line with the suppression of the STE emission through partial substitution of Sb^3+^ into [BiI_6_]^3–^ units of CABI crystal structure,^13^ which, in turn, suggests that distortion of [BiI_6_]^3–^ in the excited state produces the STE state. Moreover, the bottom of the conduction band of CABI is dominated by Bi 6p and I 5p states.^1^ We hypothesize the as-formed photogenerated free carriers or excitons on Ag–I units, which probably have higher energy than that in [BiI_6_]^3–^ units (as can be supported by varying energies at the different sites of the lattice of CABI^1,10^), relax to [BiI_6_]^3–^ on a picosecond scale to undergo the STE emission.^10,45^ Similarly, exciton energy transfer was observed from Ag- to Bi-lattice centers to produce STE emission in another silver–bismuth-halide semiconductor, Rb_4_Ag_2_BiBr_9_, but at a low temperature.^45^ Future theoretical investigations about the orbital contribution of the higher excited state (i.e., the 425 nm absorption band) in the electronic band structure of CABI and the feasibility of the energy transfer from this species to the low-lying STE state of CABI may verify our hypothesis. It should also be noted that the absence of any additional Cu_2_Ag_2_I_4_-related emissive phases (from XRD data analysis and FLIM images) in the CABI sample suggests that the nature of the observed emission is intrinsic, arising from the strong charge-carrier coupling.^10^

In conclusion, we propose an exciton energy transfer mechanism for the STE emission in CABI, i.e., photoexcited carriers from a higher energy state (which corresponds to the 425 nm absorption band) are self-trapped into the low-lying STE states and then generate the emission. Comparing PLE and TA characteristics between CABI and Cu–Ag–I-rich phases suggests that silver-based lattice sites contribute to the initial photoexcited state in CABI, although the emission pathways are not the same in both materials.

Being the front runner in the lead-free PIMs for IPVs, understanding the basis of the carrier self-trapping in CABI will direct its further optimization for various charge extraction-based applications (photovoltaics, X-ray detectors, and photoelectrochemical fuel generation) and the discovery of materials with similar optoelectronic properties. Since our proposed carrier trapping mechanism involves [BiI_6_]^3–^ units, the partial alloying of the Bi^3+^ lattice sites with smaller or larger cations or the I^–^ ions with other halide ions may alter the local structural symmetry^13,46^ and mitigate the intrinsic carrier trapping pathways, in turn improving the performance of photovoltaic devices. Our findings will stimulate novel theoretical and/or experimental investigations for optimizing low-dimensional lead-free PIMs through compositional engineering.

## Supporting Information Available

Experimental section; XRD pattern and comparison of PB and PIA bands of CABI; optical properties of CABI; evolution of XRD patterns, absorption, PL, and PLE spectra of Cu–Ag–Bi–I films with temperature; PLE and absorption spectral comparison of Cu_2_Ag_2_I_4_:Bi; TA decay curves of PB bands at 430 nm; deconvolution of PL spectrum of Cu_2_Ag_2_I_4_:Bi; TA spectra of Cu_2_Ag_2_I_4_:Bi (500 to 750 nm); Rietveld refinement outcomes (PDF)
