# Supplementary material for: Role of Self-Trapped Excitons in the Broadband Emission of Lead-Free Perovskite-Inspired Cu2AgBiI6
Source: J Phys Chem Lett. 2023 Apr 28;14(18):4192–9. doi: 10.1021/acs.jpclett.3c00439 (PMC10184165; doi:10.1021/acs.jpclett.3c00439)
Supplement: Supplementary file 1 — jz3c00439_si_001.pdf [file jz3c00439_si_001.pdf]

Supporting Information (SI)

Role of Self-Trapped Excitons in the Broadband Emission of Lead-Free Perovskite-Inspired Cu_2AgBiI_6

G. Krishnamurthy Grandhi,[1] Rakesh Dhama,[2] Noolu Srinivasa Manikanta Viswanath,[3] Ekaterina S. Lisitsyna,[4] Basheer Al-Anesi, [1] Jayanta Dana,[4] Vipinraj Sugathan,[1] Humeyra Caglayan[2], Paola Vivo*[1]*

[1]Hybrid Solar Cells, Faculty of Engineering and Natural Sciences, Tampere University, P.O. Box 541, FI-33014 Tampere University, Finland

*Email: murthy.grandhi@tuni.fi, paola.vivo@tuni.fi

[2]Tampere University, Faculty of Engineering and Natural Sciences, 33720 Tampere, Finland

[3]Division of Materials Science and Engineering, Hanyang University, 222 Wangsimni-ro, Seongdong-gu, Seoul, 04763, Republic of Korea

[4]Faculty of Engineering and Natural Sciences, Tampere University, Korkeakoulunkatu 8, 33720 Tampere, Finland

Table of Contents

Experimental section S3

Figure S1 .... S5

Figure S2 .... S6

Figure S3. .... S7

Figure S4 .... S8

Figure S5 .... S9

Figure S6 .... S10

Figure S7 .... S11

Figure S8. .... S12

Figure S9 .... S13

Table S1. Rietveld refinement parameters $Cu_2Ag_2I_4$:Bi. S14

Table S2. Refined structural parameters of $Cu_2Ag_2I_4$:Bi. S14

Experimental section

Materials

Copper iodide (CuI), Bismuth iodide (BiI_3), and dimethylsulfoxide (DMSO) were purchased from Sigma-Aldrich. Silver iodide (AgI) and Dimethylformamide (DMF) were purchased from Alfa Aesar.

CABI and $Cu_2Ag_2I_4$:Bi film fabrication

The precursor solution was obtained by mixing CuI (238.1mg), AgI (241.8 mg), and BiI_3 (517.1mg), in 2 ml DMSO: DMF (3:1) mixture and then heated at 150 °C for 40–45 min inside glove box until it became clear and transparent. Cu-Ag-Bi-I (CABI and $Cu_2Ag_2I_4$:Bi) films on glass were deposited spin-coating at 3000 rpm for 1 min and then immediately annealing in the air for 50 minutes at 50 °C and then for 4 minutes at 75−330 °C, as demonstrated in Figure S4.

Characterization methods

XRD patterns were collected using the Malvern Panalytical Empyrean multipurpose diffractometer with a Cu K_α X-ray source (λ = 0.15418 nm). The films were scanned over 10°–60° with a step size of 0.026°. The crystal structures were drawn using visualization system for the electronic and structural analysis (VESTA) program[1]. The top view SEM images of CABI and $Cu_2Ag_2I_4$:Bi films were obtained using a field emission scanning electron microscope (FE-SEM, Zeiss ULTRA plus, Carl Zeiss, Germany) at 3kV. The elemental composition of the films was extracted with the help of EDS spectroscopy (Oxford Instruments X-MaxN 80 EDS) in combination with the FE-SEM. UV-Visible absorption spectra of the films were measured using a Shimadzu UV-1800 absorption spectrometer (dual-beam grating), Shimadzu Corporation, Japan. Steady-state PL measurements were performed on FLS1000 spectrofluorometer from Edinburgh Instruments, UK. Absolute PL QY was determined using an integrating sphere (in "powder" mode) for CABI and $Cu_2Ag_2I_4$:Bi films under 405 nm excitation. Time-correlated single photon counting (TCSPC) measurements for the TRPL decay were performed on a device equipped with a Picoharp 300 controller, and a PDL 800-B driver employed for the excitation. The signal detection in the 90° configuration was executed by a Hamamatsu R3809U-50 microchannel plate photomultiplier. Fluorescence lifetime images were acquired using a fluorescence lifetime microscope MicroTime-200 (PicoQuant,

Germany) coupled to the inverted microscope Olympus IX-71 (Olympus, Japan). The CABI samples were applied to microscope glass slides and imaged with a 60 × water objective having NA 1.2. A pulsed diode laser LDH-P-C-405 (PicoQuant) emitting at 405 nm with a 60 ps resolution was used for the fluorescence excitation. The emission was monitored using 430 nm long pass filter. The SymPhoTime 64 software was used to calculate the lifetime map images. In FLIM images (Figure 2a, c) the colors present fast lifetimes (τ_{fast}) or mean arrival times of the emitted photons detected in each pixel. To gain information on the actual lifetime changes, regions of interest (ROIs) were selected, and the corresponding fluorescence decays were extracted (see Figure 2b). The fluorescence decay curves were fitted by applying iterative least-squares method to the sum of exponents in the equation $I(t, \lambda) = \sum_i a_i(\lambda) e^{-t/\tau_i}$ where τ_i is the lifetime and $\alpha_i(\lambda)$ is the amplitude (pre-exponential factor) and the amplitude weighted average fluorescence lifetimes were calculated as $< \tau >_\alpha = (\sum_i a_i \tau_i)/(\sum_i a_i)$. For transient absorption measurements, ultrafast time-resolved pump-probe spectroscopy was performed using an amplified Ti: sapphire laser system equipped with an optical parametric amplifier (OPA). This system produced 100 fs pulses at 1.00 kHz with a center wavelength of 800 nm. Most of the output (90%) was sent to OPA to generate tunable pump pulses in the UV-Visible to near-infrared spectral regions to excite the samples at desired wavelength. The remaining 10% of output power travels through a delay-line to enable controlled time difference between pump and probe pulses and converts into a broadband probe beam to interrogate the sample at the normal incidence in transmission mode. At the same time, the chopper-modulated pump pulse is spectrally as well as temporally overlapped with the probe beam on the sample. At the same time, the detector is triggered to detect every probe pulse and calculate the absorption spectrum. Repetition rates of the pump and probe beams turn out to be 500 Hz and 1 kHz, respectively. Therefore, the effect of the pump beam will be observed only in one of the two consecutive probe beams. The fully automatic optical delay line in our set-up can take a minimum step size of 2.8 fs. For example, data can easily be acquired at a delay of 556 ± 3 fs.

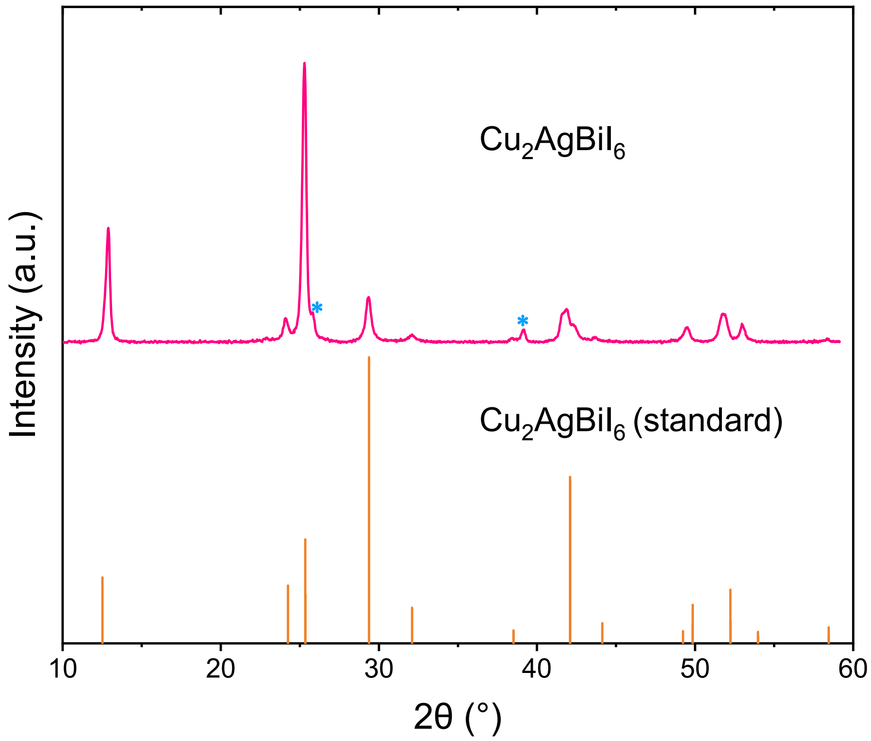

Figure S1. The XRD pattern of a CABI film along with the corresponding standard pattern. The peaks marked with the asterisk symbol denote the XRD reflections corresponding to the Cu-Bi-I impurities.[2,3]

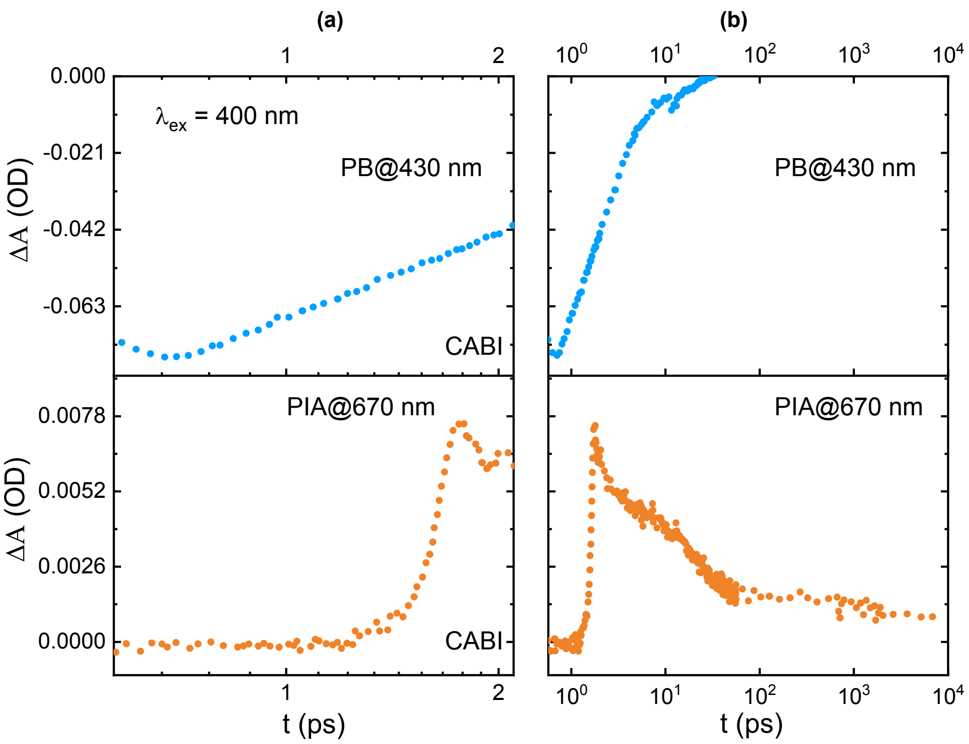

Figure S2. Comparison of the TA decay curves of the PB (at 430 nm) and PIA (at 670 nm) bands of CABI (see Figures 1d and 1e) up to (a) 2 ps and (b) a few ns.

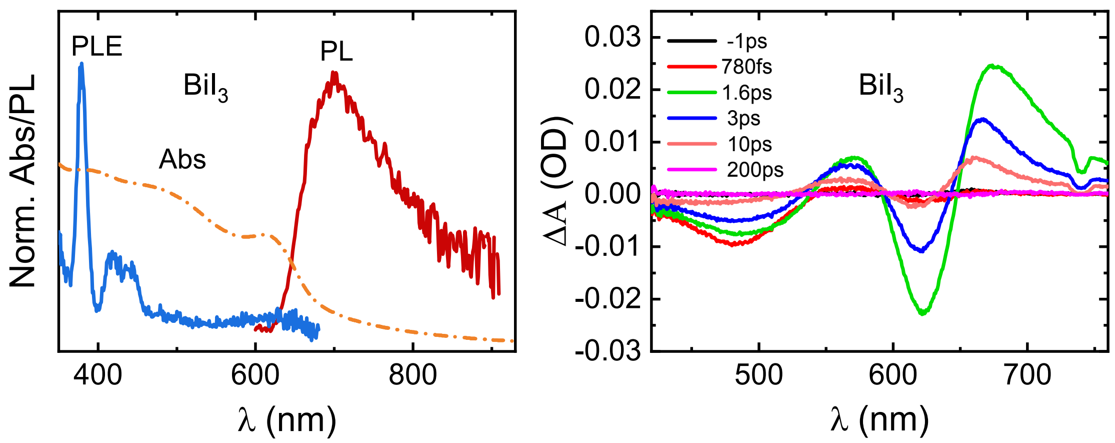

Figure S3. Absorption, PL, and PLE spectra (left) and TA spectra (right) of BiI_3 films. The excitation wavelength for recording PL and TA spectra is 400 nm.

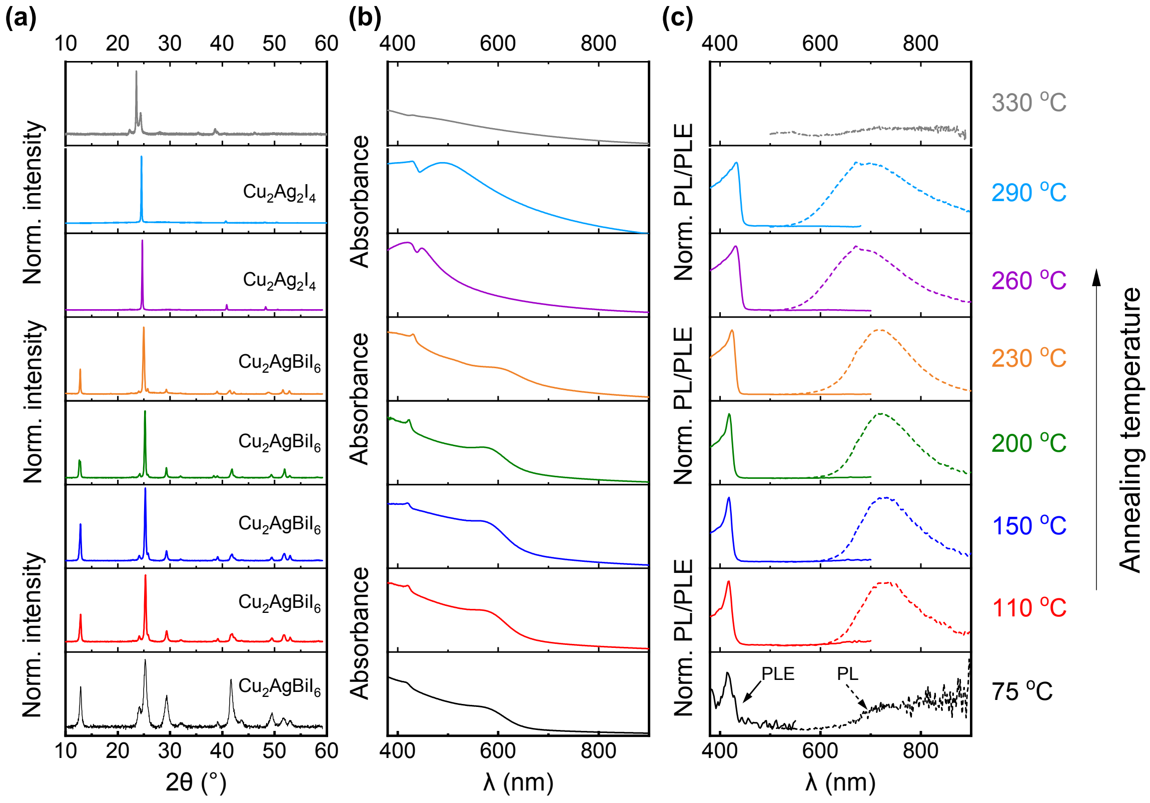

Figure S4. (a) The XRD patterns, (b) absorption spectra, and (c) PL and PLE spectra of Cu-Ag-Bi-I films fabricated in the annealing temperature range of 75−330 °C.

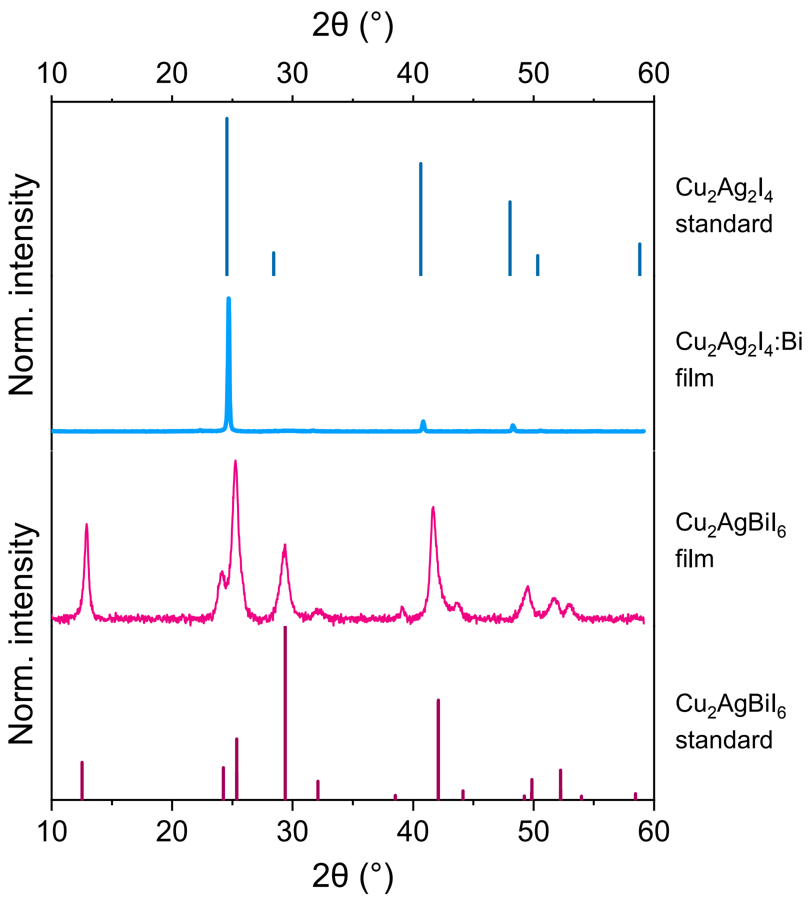

Figure S5. XRD patterns of CABI and $Cu_2Ag_2I_4$:Bi films along with the standard patterns of CABI and $Cu_2Ag_2I_4$.

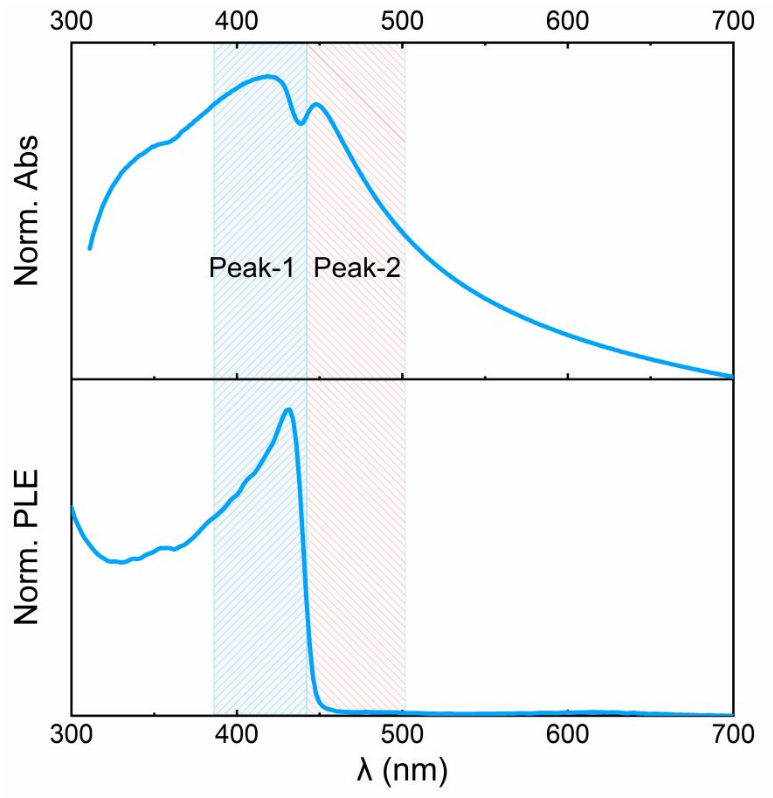

Figure S6. Comparison of the absorption and PLE spectra of $Cu_2Ag_2I_4$:Bi film.

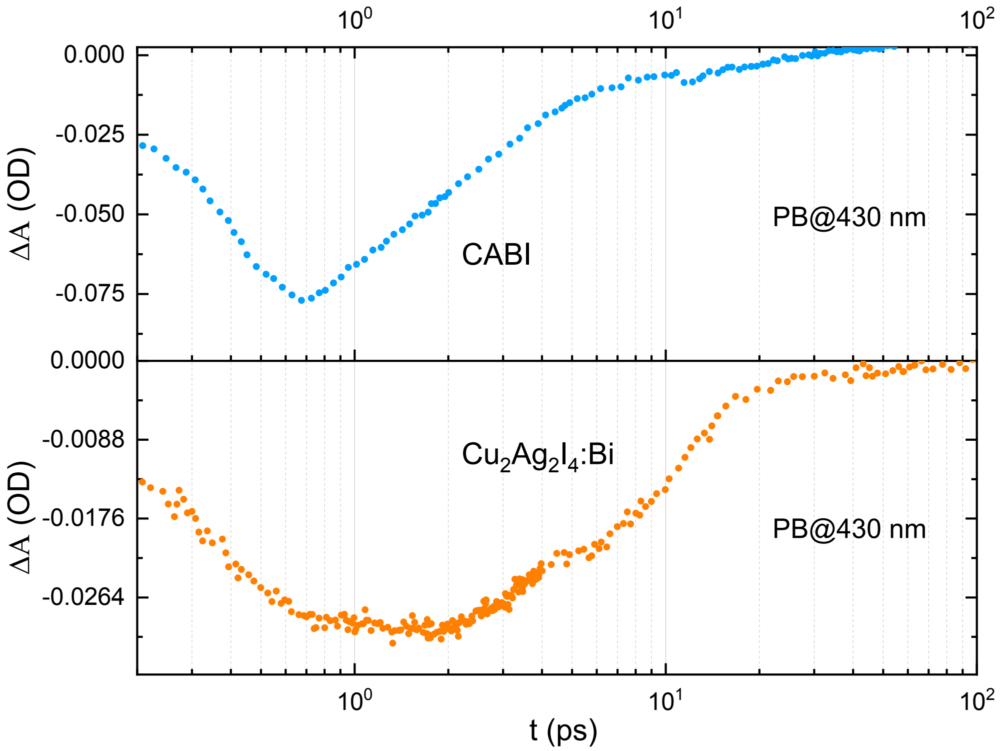

Figure S7. TA decay curves of PB bands at 430 nm for CABI (top) and $Cu_2Ag_2I_4$:Bi (bottom) films, excited at 400 nm.

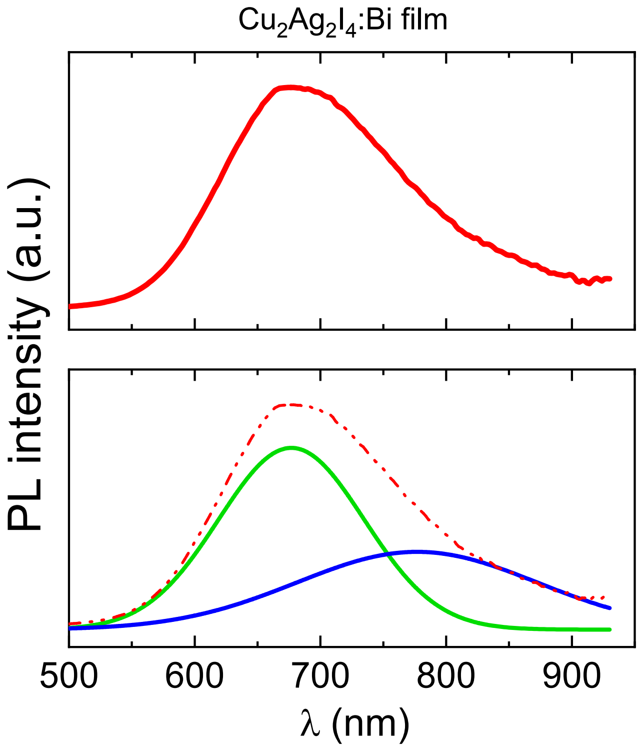

Figure S8. (a) The PL spectrum of $Cu_2Ag_2I_4$:Bi (excited at 400 nm) with inhomogeneous broadness on the two ends of the spectrum. (b) The deconvolution of the PL spectrum in (a) into two gaussian profiles.

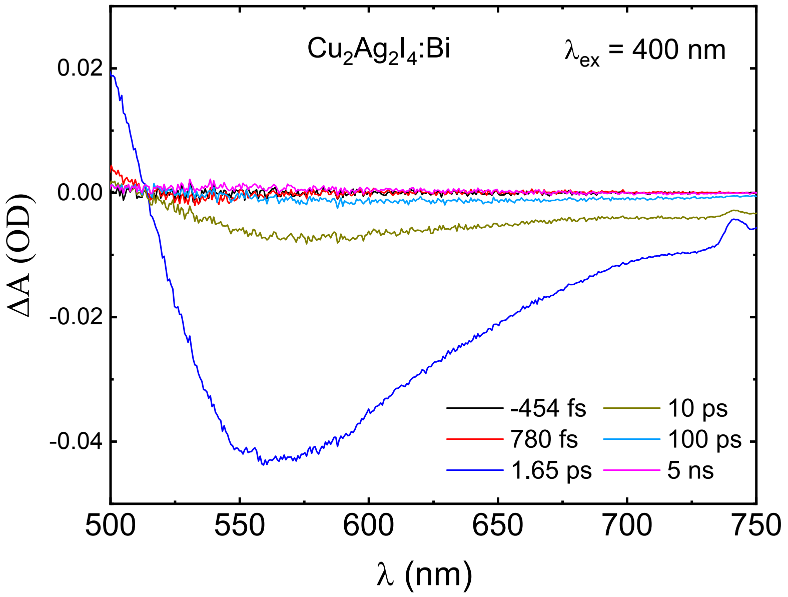

Figure S9. The TA spectra of $Cu_2Ag_2I_4$:Bi film in the 500 to 750 nm probe range, excited at 400 nm.

Table S1. Rietveld refinement parameters $Cu_2Ag_2I_4$:Bi obtained from the powder diffraction data at room temperature.

The numbers in parentheses are the estimated standard deviations of the last significant figure.

Compound	$Cu_2Ag_2I_4$:Bi
Space group	$F\bar{4}3m$
Structure	Cubic
$a=b=c$/Å	6.28(7)
V/Å^3	247.54(5)
R_{wp}	1.8%
χ^2	1.2

Table S2. Refined structural parameters of $Cu_2Ag_2I_4$:Bi.

The numbers in parentheses are the estimated standard deviations of the last significant figure.

Atom	Wyckoff position	x	y	z	Occupancy	100xU_{iso}(Å)^2
Cu	4*a*	0	0	0	0.50	0.0186(23)
Ag	4*a*	0	0	0	0.46	0.0186(23)
Bi	4*a*	0	0	0	0.04	0.0186(23)
I	4*c*	0.25	0.25	0.25	0.92	0.2822(17)

References

(1) Momma, K.; Izumi, F. VESTA 3 for Three-Dimensional Visualization of Crystal, Volumetric and Morphology Data. *J. Appl. Crystallogr.* **2011**, *44*, 1272–1276.

(2) Sansom, H. C.; Longo, G.; Wright, A. D.; Buizza, L. R. V; Mahesh, S.; Wenger, B.; Zanella, M.; Abdi-Jalebi, M.; Pitcher, M. J.; Dyer, M. S. Highly Absorbing Lead-Free Semiconductor Cu_2AgBiI_6 for Photovoltaic Applications from the Quaternary CuI–AgI–BiI_3 Phase Space. *J. Am. Chem. Soc.* **2021**, *143* (10), 3983–3992.

(3) Grandhi, G. K.; Al-Anesi, B.; Pasanen, H.; Ali-Löytty, H.; Lahtonen, K.; Granroth, S.; Christian, N.; Matuhina, A.; Liu, M.; Berdin, A.; Pecunia, V.; Vivo, P. Enhancing the Microstructure of Perovskite-Inspired Cu-Ag-Bi-I Absorber for Efficient Indoor Photovoltaics. *Small* **2022**, *18*, 2203768.
